# Enterobactin Deficiency in a Coliform Mastitis Isolate Decreases Its Fitness in a Murine Model: A Preliminary Host–Pathogen Interaction Study

**DOI:** 10.3389/fvets.2020.576583

**Published:** 2020-11-09

**Authors:** Niels Vander Elst, Koen Breyne, Jonas Steenbrugge, Amanda Jane Gibson, David George Emslie Smith, Pierre Germon, Dirk Werling, Evelyne Meyer

**Affiliations:** ^1^Laboratory of Biochemistry, Department of Pharmacology, Toxicology and Biochemistry, Faculty of Veterinary Medicine, Ghent University, Merelbeke, Belgium; ^2^Molecular Neurogenetics Unit, Department of Neurology and Center for Molecular Imaging Research, Massachusetts General Hospital and Harvard Medical School, Boston, MA, United States; ^3^Centre of Excellence for Bovine TB, Institute of Biology, Environmental and Rural Sciences, Aberystwyth University, Aberystwyth, United Kingdom; ^4^Department of Pathobiology and Population Sciences, Royal Veterinary College, Hatfield, United Kingdom; ^5^School of Engineering and Physical Sciences, Institute of Biological Chemistry, Biophysics and Bioengineering, Heriot-Watt University, Edinburgh, United Kingdom; ^6^INRAE, UMR ISP, Université François Rabelais de Tours, Nouzilly, France

**Keywords:** *E. coli* mastitis, enterobactin, iron scavenging, siderophores, lipocalin 2 (also known as NGAL)

## Abstract

Iron is an essential nutrient for bacterial growth. Therefore, bacteria have evolved chelation mechanisms to acquire iron for their survival. Enterobactin, a chelator with high affinity for ferric iron, is secreted by *Escherichia coli* and contributes to its improved bacterial fitness. In this preliminary study, we evaluated enterobactin deficiency both *in vitro* and *in vivo* in the context of *E. coli* mastitis. Firstly, we showed that expression of lipocalin 2, a protein produced by the host that is able to both bind and deplete enterobactin, is increased upon *E. coli* infection in the cow's mastitic mammary gland. Secondly, we demonstrated *in vitro* that enterobactin deficiency does not alter interleukin (IL)-8 expression in bovine mammary epithelial cells and its associated neutrophil recruitment. However, a significantly increased reactive oxygen species production of these neutrophils was observed. Thirdly, we showed there was no significant difference in bacterial *in vitro* growth between the enterobactin-deficient mutant and its wild-type counterpart. However, when further explored in a murine model for bovine mastitis, the enterobactin-deficient mutant vs. the wild-type strain revealed a significant reduction of the bacterial load and, consequently, a decrease in pro-inflammatory cytokines (IL-1α,−1β,−4,−6, and−8). A reduced neutrophilic influx was also observed immunohistochemically. These findings therefore identify interference of the enterobactin iron-scavenging mechanism as a potential measure to decrease the fitness of *E. coli* in the mastitic mammary gland.

## Introduction

Iron (Fe) is an essential micronutrient and co-factor in several oxidation–reduction reactions required for basic cell metabolism, e.g., DNA stability and cell cycle control (1, 2). In mammals, iron availability is tightly regulated by host iron-binding proteins such as transferrin, ferritin, and lactoferrin. The latter is specifically secreted in milk in order to limit bacterial growth (3, 4). However, bacteria secrete high affinity iron-binding siderophores to circumvent iron restriction by the host. Enterobactin (Ent) is a prototypic siderophore that is highly conserved in *Escherichia coli*, being optimally structured for binding ferric iron (5–7). For its synthesis, Ent relies on the conversion of amino acid precursors by the non-ribosomal peptide synthetase EntA which catalyzes an early step in Ent biosynthesis (6, 8, 9). The production of siderophores like Ent is particularly induced under low iron level conditions, aiding bacteria in their competition with the host for scarcely available iron (1, 10). The role of Ent is especially well-studied in the inflamed gut in which iron-bound Ent from intestinal *E. coli* clearly contributes to pathogen growth and immune resistance during disease development (11–13). Therefore, Ent and other siderophores have been identified as potential preventive or therapeutic targets in general and have shown some applicability, also to bovine mastitis pathogens (14, 15). Bovine mastitis, an inflammation of the cow's udder, is currently the most prevalent production disease in the dairy industry and is frequently caused by *E. coli* (16, 17). *Escherichia coli* is an environmental pathogen that takes advantage of poor hygiene conditions and is best controlled with appropriate farm and animal management measures (18). However, full eradication of *E. coli* mastitis even with these appropriate measures implemented proves impossible (19).

Bacterial siderophores have been associated with lipocalin 2 (LCN2) released by host immune cells, primarily polymorphonuclear neutrophilic granulocytes (PMN), as part of their immune response against propagating pathogens (11–13, 20). LCN2 is able to bind and deplete both iron-bound and iron-lacking bacterial siderophores, thus further preventing iron uptake by bacteria (12, 21, 22). These LCN2-producing PMN are attracted to the infected mammary gland by the chemotactic molecule interleukin (IL)-8 that is secreted by the bovine mammary epithelium to enhance phagocytosis and reactive oxygen species (ROS) production of PMN to clear the infection present (23, 24).

However, the interplay between bacterial Ent and the host's immune system remains poorly studied in the context of *E. coli* mastitis (25). For this purpose, limited *in vitro* and *in vivo* studies were combined to preliminarily investigate the involvement of iron availability, innate immunity, and a local bacterial infection.

## Materials and Methods

### Selection of *E. coli* Strains

Three *E. coli* strains were selected: P4, 1303, and MG1655. The P4 strain consisted of a wild-type bovine mastitis isolate (P4) as well as its genetically modified EntA-deficient counterpart (P4 Δ*entA*) unable to produce enterobactin. To demonstrate a strain-independent effect, *E. coli* strains 1303 and MG1665 were included additionally. *Escherichia coli* 1303 is bovine mastitis-derived, and *E. coli* MG1655 is a non-pathogenic *E. coli* K-12 derivative (26, 27). All *E. coli* strains were grown in Dulbecco's modified Eagle's medium (DMEM)/F12 (Thermo Fisher Scientific) at 37°C and 5% CO_2_.

### Quantitative Polymerase Chain Reaction of LCN2 mRNA in Udder Tissue of Experimentally *E. coli*-Infected Dairy Cows

The experimental procedures on the cows were executed at the Clinic for Ruminants in Munich under ethical approval by the regional government ethics committee of upper Bavaria (approval number 55.2-1-54-2531-108-05). Five mid-lactating Holstein Friesian cows were experimentally infected in one quarter with either 500 colony-forming units (CFU) of *E. coli* mastitis isolate 1303 or were sham-inoculated (28). Prior to the infection, milk samples of all udder quarters confirmed normal somatic cell counts (50,000 somatic cells/ml) and the absence of mastitis pathogens. Twelve hours post-infection (h.p.i.), all inoculated animals displayed clinical mastitis in the affected quarters. Animals were humanely euthanized 24 h.p.i. following the recommended guidelines of the American Veterinary Medical Association (“AMVA Guidelines for the Euthanasia of Animals”), and ~100 mg of lobulo-alveolar udder tissue was snap frozen in liquid nitrogen immediately after euthanasia. RNA was extracted from the udder tissue of the *E. coli*-infected cows using TRIzol (Invitrogen). Reverse transcription of 100 ng total RNA into cDNA was performed using iScript Reverse Transcription Supermix (Bio-Rad). Subsequently, iQ SYBR Green Supermix (Bio-Rad) was used for quantitative polymerase chain reaction (qPCR) with the primers described in Supplementary Table 1. Expression of two reference genes PPIA and 18S, according to MIQE guidelines, was used for normalization, and Genex macro (Bio-Rad) then calculated the final gene expression in infected vs. uninfected udder quarters from the same cow (29).

### IL-8 Expression of *E. coli*-Exposed Bovine Mammary Epithelial Cells

Two bovine mammary epithelial cell lines (boMECs), MAC-T and PS, were exposed to either *E. coli* P4, P4 Δ*entA*, 1303, or MG1665 as described (30). Briefly, boMECs were seeded to flat microtiter plates to contain 10^5^ cells/well in assay medium DMEM/F12 (Thermo Fisher Scientific) and grown to confluence at 37°C and 5% CO_2_ for 72 h (31). Prior to infection of boMECs, *E. coli* strains were inoculated into DMEM/F12 and grown standing at 37°C for 2–4 h to reach OD_600_ = 0.4 before diluting to 10^6^ CFU/ml. After co-incubation of each *E. coli* with boMECs for 3 h, supernatants were collected. *Escherichia coli* were removed by washing with Hanks' balanced salt solution (HBSS) (Gibco) and media replenished supplemented with 50 μg/ml gentamycin (Sigma) for a further 21 h. Supernatants were additionally collected at 24 h. Secreted IL-8 levels were then measured in the supernatant of both exposed boMECs by enzyme-linked immunosorbent assay (ELISA) as described (32).

### Isolation and Migration of and Reactive Oxygen Species Production by PMN After Co-culture With *E. coli*-Exposed boMECs

Migration of PMN was evaluated by a transwell assay. PMN were isolated from peripheral bovine blood by density centrifugation and flash lysis as described (33). PMN purity was assessed by microscopy after Diff-Quik (Reagena) staining. Isolations that were at least 90% pure were subsequently used for migration assays. The latter were performed using transwell inserts for 24-well tissue culture plates containing a pore size of 2 μm (Greiner). BoMECs were introduced into 24-well plates at a concentration of 10^5^ cells/well and allowed to adhere overnight. Cells were washed and media replaced with *E. coli* isolates at a multiplicity of infection (MOI) of 10^5^ CFU/well, arbitrarily set as 1 (MOI = 1). Media alone and recombinant bovine IL-8 (Kingfisher Biotech) at 5 μg/ml served as the negative and positive control, respectively. PMN were immediately added to the inner transwell at 10^6^ cells/well and subsequently incubated during 24 h. Migration was assessed 3 and 24 h after *E. coli* exposure using modified counting chambers (Immune Systems) as described (34). Reactive oxygen species production of PMN was investigated by culturing isolated PMN in HBSS either in the presence of P4 or P4 Δ*entA* heat-killed *E. coli*. Reactive oxygen species was measured as described (35). Briefly, production of ROS by PMN was measured by oxidation of 2,7-dichlorofluorescein diacetate (DCFH-DA) (Sigma) to fluorescent 2,7-dichlorofluorescein (DCFH) at 2 h post-exposure in black microtiter plates. Zymosan-stimulated and unstimulated PMN served as positive and negative control, respectively. Relative fluorescence units were measured using a spectrophotometer (Tecan M200) at 485 nm excitation and 530 nm emission wavelengths, while cells were incubated at 37°C and 5% CO_2_.

### *In vitro* Growth Curves of *E. coli* P4 and *E. coli* P4 Δ*entA*

An overnight bacterial culture was diluted 1:100 in DMEM/F12 (Thermo Fisher Scientific) and vortexed. Subsequently, 200 μl thereof was added to wells in a 96-well plate. This 96-well plate was thereafter incubated at 37°C and the optical density was measured at 600 nm (OD_600_) every hour during 8 h.

### Mouse Model for Bovine *E. coli* Mastitis and Processing of the Harvested *E. coli*-Infected Mammary Glands

All experimental procedures on mice were executed at the Faculty of Veterinary Medicine of Ghent University (Merelbeke, Belgium) and with approval of the ethics committee (Approval Number 2015/127). In brief, female C57BL/6 and CD-1 mice (purchased from Envigo) were coupled with male mice. Ten days post-partum, the lactating dams were intraductally inoculated with a 32-gauge pediatric needle in the fourth abdominal mammary gland pair after properly disinfecting the teat. Intraductal inoculations were performed with an inoculum dose of ~250 CFU at 1 h post-weaning of either P4 or P4 Δ*entA* in 100 μl phosphate buffered saline (PBS). This inoculation was performed under general gas anesthesia, using a mixture of oxygen and isoflurane at 2–3% for induction and 1–1.5% for maintenance. The long-acting analgesic buprenorphine (Vetergesic, Patheon UK Ltd., Swindon, UK) was administered at 10 μg/kg intraperitoneally for post-surgical pain relief. At 24 h.p.i., the inoculated mice were sedated with a cocktail of 100 mg/kg ketamine (Ketamidor, Ecuphar nv/sa, Belgium) and 10 mg/kg xylazine (Xylazini Hydrochloridum, Val d'Hony-Verdifarm, Belgium) and subsequently euthanized by cervical dislocation to harvest the mammary glands. In order to quantify the bacterial loads (expressed as CFU), the isolated mammary glands were homogenized using TissueRuptor (Qiagen) and serially diluted in PBS, followed by plating on tryptic soy agar (Oxoid). To compensate for the different volumes of the harvested tissues, mammary glands were weighed and bacterial load was expressed as CFU/g tissue.

The host response to the experimental mammary gland infection was investigated by determining the levels of LCN2 and selected inflammatory cytokines in mammary gland lysates obtained by mixing the homogenates with 300 μl lysis buffer containing protease inhibitors. Quantification of LCN2 was performed by ELISA (Bio-Techne, Minneapolis, MN, USA). Levels of the selected cytokines IL-1α,−1β,−4,−6, and−8 were determined by multiplex assay (Luminex, Thermo Fisher Scientific). Neutrophil influx was evaluated by immunohistochemical staining for the neutrophil marker Ly6G (anti-Ly6G-APC, clone 1A8, Miltenyi Biotec) on paraffin sections of the infected mouse mammary glands at 24 h.p.i. Deparaffinized and hydrated tissue slides were incubated in citrated buffer at pH 6.0 (10 mM tri-sodium citrate and 0.05% Tween-20) in a decloacking chamber under pressure at 95°C for 30 min. Following peroxidase blocking [3% H_2_O_2_ in methanol for 10 min at room temperature (RT)], sections were incubated with primary rat anti-mouse Ly6G antibody for 1 h at RT. Rat-on-mouse HRP-Polymer (Biocare Medical) served as secondary antibody on the tissue sections for 30 min at RT. Detection of the staining was performed by applying a buffer with 3,3′-diaminobenzidine on the tissue sections for 10 min at RT. For microscopic evaluation of this immunohistochemical staining, the tissue sections were rehydrated and mounted with a cover glass.

### Statistical Analysis

Data were analyzed using GraphPad Prism (version 5.00.288) to calculate *P*-values and determine statistically significant differences (*P* < 0.05). D'Agostino-Pearson was used to check for normally distributed data. If needed, data underwent a log10 transformation, leading to a normal distribution of the data. Two groups were compared with unpaired *t*-tests, and multiple groups with analysis of the variance (ANOVA) and Tukey's *post hoc*-test.

## Results

### Expression of Host LCN2 Is Increased in the *E. coli*-Infected Mammary Gland

**Figure 1:**
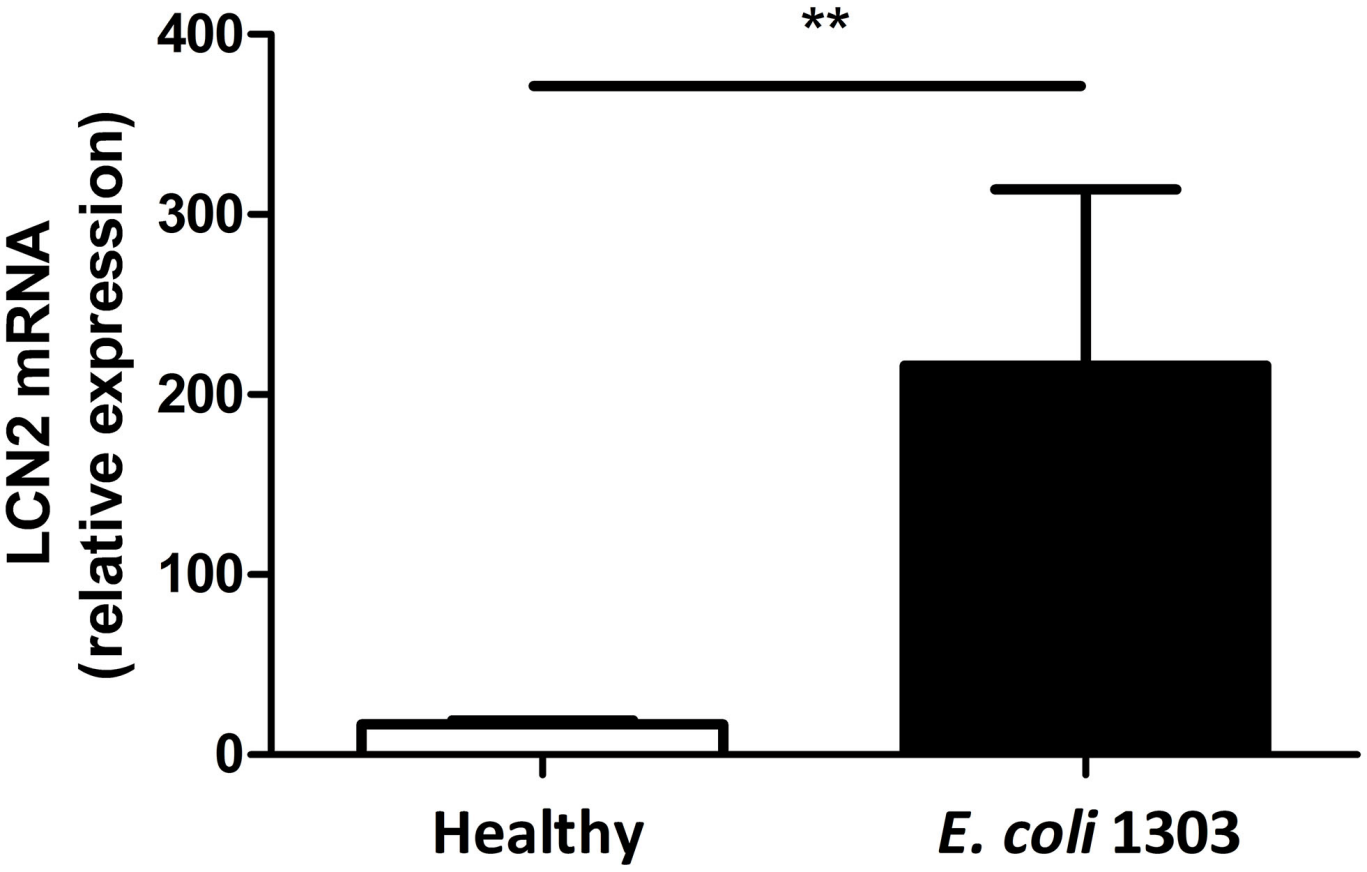
mRNA lipocalin 2 (LCN2) relative expression calculated by the ΔΔCt method taking as reference genes PPIA and 18S in udder quarters 24 h.p.i. with 500 colony-forming units (CFU) *E. coli* 1303 compared with sham-inoculated udder quarters (*n*_quarters_ = 5 per condition). Data are presented as means ± standard error of the mean. **P < 0.01.

To confirm that iron is sequestered in the cow's mastitic mammary gland by the host's immune system, Holstein Friesian cows were experimentally infected with *E. coli* 1303 and LCN2 expression was determined. qPCR analysis revealed that LCN2 expression in the *E. coli-*infected bovine mammary gland tissue was indeed significantly increased compared with sham-inoculated quarters (*P* < 0.01; Figure 1).

Since our group previously observed the Ent expression system is commonly present in mastitis-causing *E. coli* (36) and the role of LCN2 includes sequestration of bacterial Ent, this observation in the target species raises a subsequent question: How is the host's immune response, i.e., chemokines produced by boMECs, PMN migration, and activation, affected in the presence and absence of bacteria producing Ent? Therefore, a bovine mastitis-derived *E. coli* strain, i.e., P4, was made deficient in the EntA gene, and Ent deficiency was preliminarily determined *in vitro* on the previously mentioned cell types.

### IL-8 Secretion Is Not Altered in BoMECs by Bacterial Ent Depletion

**Figure 2:**
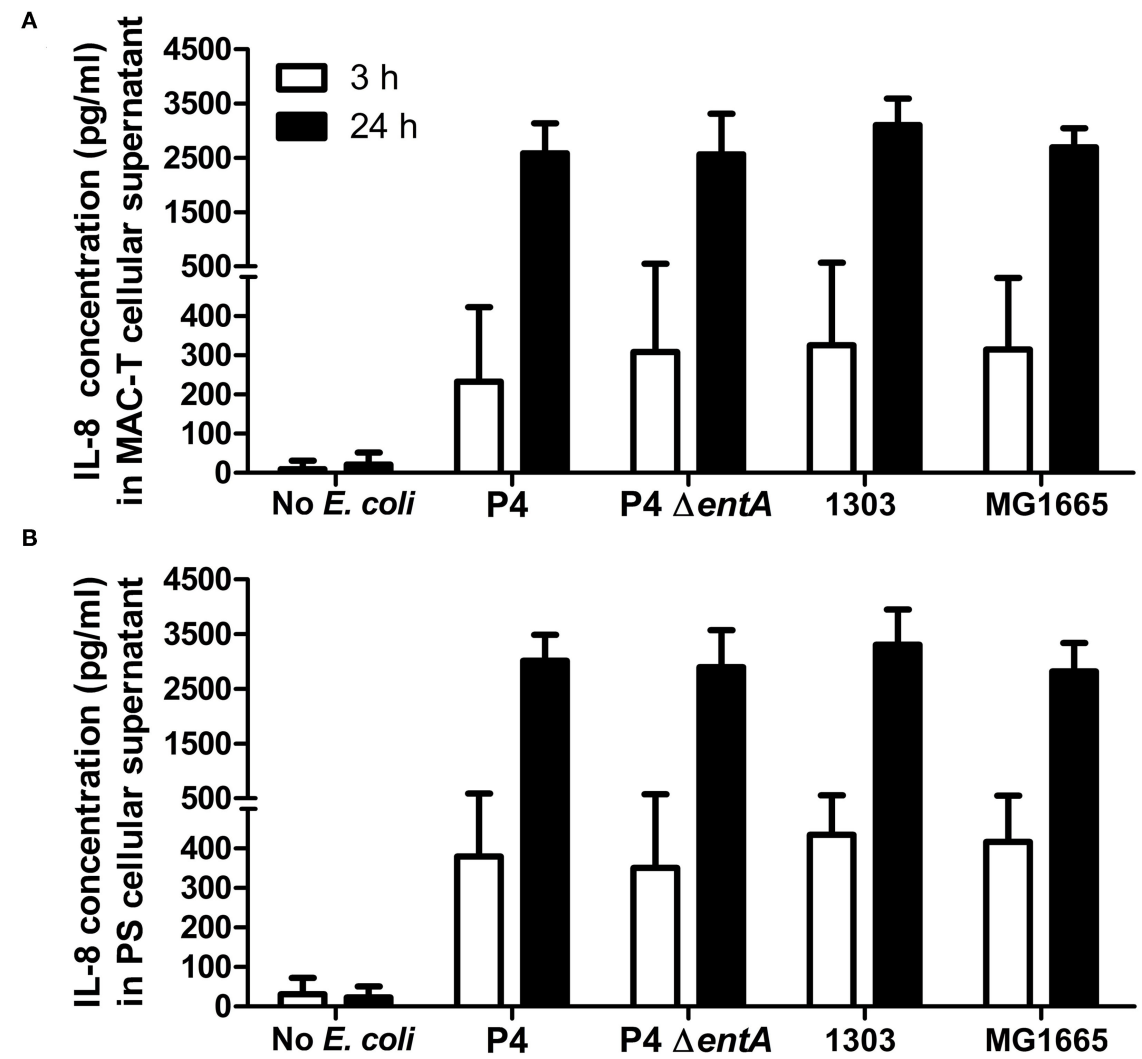
Secreted interleukin (IL)-8 concentrations by bovine mammary epithelial cell lines (boMECs) as measured in the supernatant of bovine MAC-T **(A)** and PS **(B)** cells at both 3 and 24 h after co-incubation with *E. coli* P4 Δ*entA* vs. P4 and two additional *E. coli* strains, i.e., 1303 and MG1655. Each cell type and incubation time had six replicates (*n* = 6). Data are presented as means ± standard error of the mean.

IL-8 secretion, a key inflammatory marker of mastitis, was determined as a first parameter in two boMEC lines [i.e., MAC-T (Figure 2A) and PS (Figure 2B)] upon incubation with *E. coli* P4 and its enterobactin-deficient counterpart: P4 Δ*entA*. BoMECs were incubated either with or without bacteria for 3 or 24 h. Untreated BoMECs showed very low levels of IL-8 secretion after 3 h and no increase in their IL-8 secretion over time at 24 h (Figure 2). The IL-8 secretion of boMECs incubated with *E. coli* P4 Δ*entA* vs. *E. coli* P4 did not differ significantly (*P* > 0.05; Figure 2). To confirm that this single observation was P4 strain-independent, boMECs were additionally incubated with two other *E. coli* strains, i.e., the bovine mastitis-derived *E. coli* 1303 and the non-pathogenic, human-derived *E. coli* MG1655. Both strains induced secretion of similar amounts of IL-8 compared with P4 and P4 Δ*entA* (Figure 2). It was also observed that increased incubation times from 3 to 24 h resulted in higher concentrations of secreted IL-8 in both boMEC lines and with all *E. coli* tested, but no differential secretion was observed upon Ent depletion.

### Bacterial Ent Depletion Does Not Alter PMN Migration but Influences ROS Production

Secondly, to investigate whether the unaltered IL-8 expression levels did not affect PMN functionality, PMN migration and ROS production of PMN were determined under the same experimental conditions.

**Figure 3:**
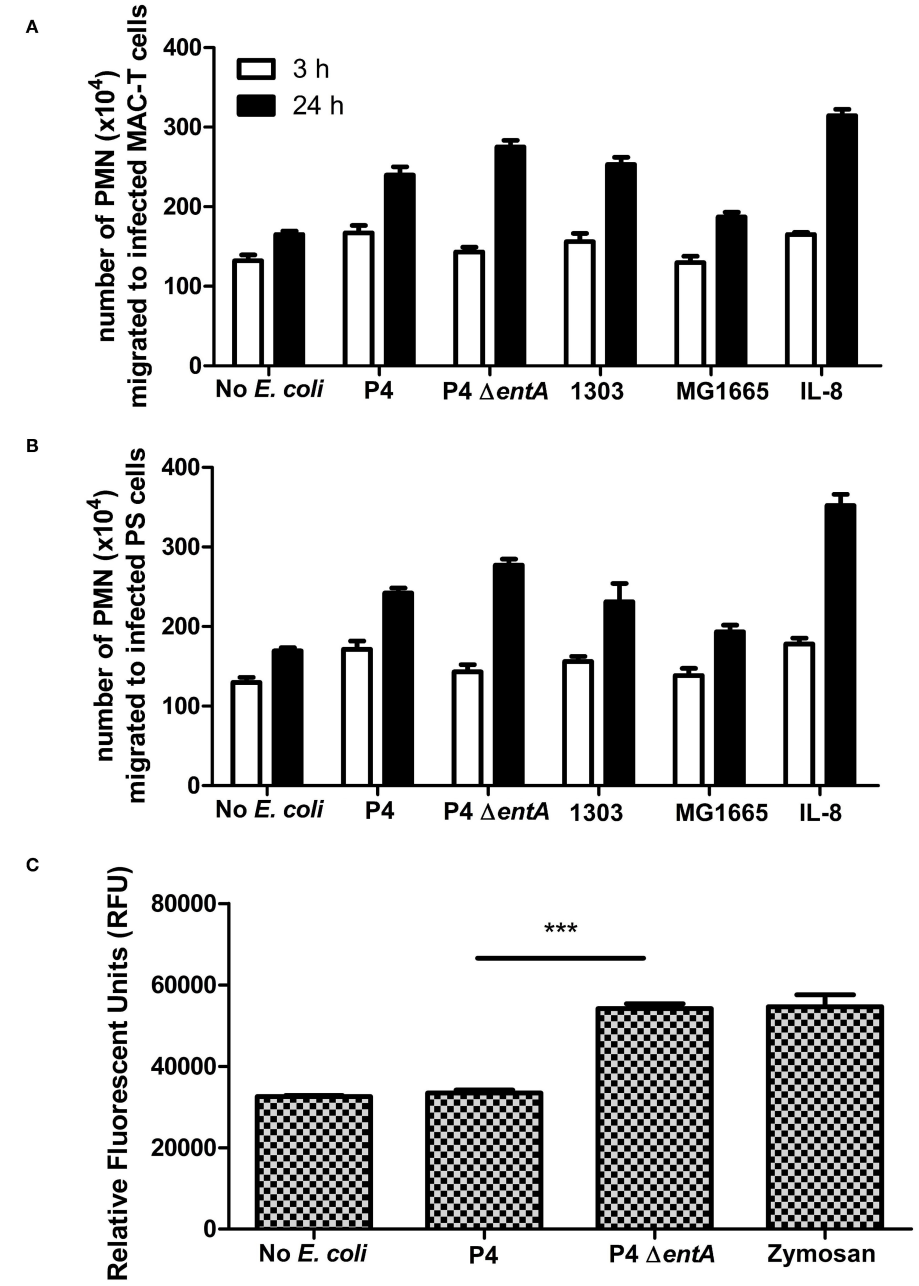
**(A,B)** Transwell migration of bovine blood-derived polymorphonuclear neutrophilic granulocytes (PMN) to boMEC lines MAC-T and PS incubated either 3 or 24 h with *E. coli* P4 Δ*entA* vs. P4 and two additional *E. coli* strains: 1303 and MG1655. Recombinant IL-8 and the absence of bacteria were used as positive and negative control, respectively. **(C)** Reactive oxygen species (ROS) production in relative fluorescence units (RFU) by bovine blood PMN measured at 485 nm excitation and 530 nm emission wavelengths for 120 min following incubation with *E. coli* P4 Δ*entA* vs. P4. Zymosan-stimulated PMN served as a positive control and were compared with unstimulated PMN as a negative control. Each cell type and incubation time had at least three replicates (*n* ≥ 3). Data are presented as means ± standard error of the mean. ***P < 0.001.

BoMECs were incubated either with or without the different *E. coli* strains, and PMN migration was subsequently evaluated using a transwell assay. Similarly as for IL-8 secretion, PMN migration did not differ significantly between P4 Δ*entA* and P4 (*P* > 0.05; Figures 3A,B). Longer incubation times resulted in a higher number of migrated PMN, but again no differential effect of Ent depletion was observed. The lack of differences was not due to a functional impairment of PMN, as PMN migrated strongly in the presence of added bovine IL-8 (positive control). After 24 h, this positive control had the highest number of migrated PMN. Furthermore, all wells containing either treated boMEC or IL-8 contained more migrated PMN compared with the negative control (untreated boMEC), although no statistically significant difference was observed either between the *E. coli* strains tested and the controls on both boMEC cell lines and as a result of increased incubation times (*P* > 0.05; Figures 3A,B). Finally, PMN migrated in general less when the wells contained only the bacterial isolates compared to incubation with *E. coli*-exposed boMECs (data not shown).

In order to determine if Ent affected oxidative antimicrobial responses of PMN, ROS production in response to P4 and P4 Δ*entA* was measured. Interestingly, measuring fluorescence intensity as marker for ROS production was significantly increased in bovine PMN incubation with P4 Δ*entA* compared with P4 (*P* < 0.001; Figure 3C). The level of ROS production of PMN in response to P4 Δ*entA* was similar to the ROS production measured in zymosan-stimulated PMN, which served as a positive control. Unstimulated and P4-stimulated PMN produced similar amounts of ROS, indicating that wild-type P4 might interfere with ROS production (Figure 3C).

### *E. coli* P4 and P4 Δ*entA* Show a Similar *in vitro* Growth

**Figure 4:**
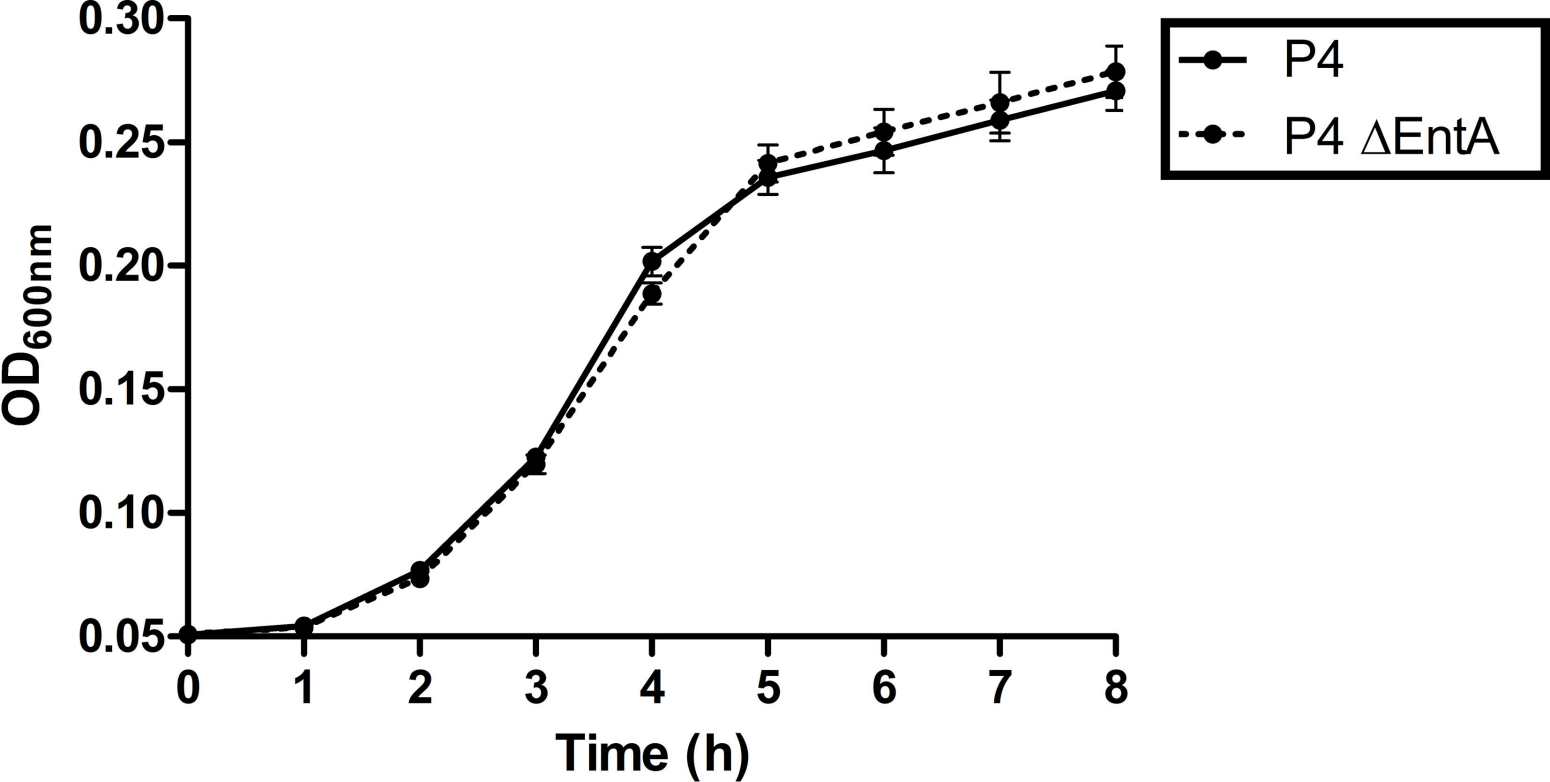
Growth curves of *E. coli* P4 and P4 Δ*entA* in DMEM/F12. Overnight bacterial cells were diluted 1:100 and 200 μl of the diluent was added to 12 (*n* = 12) wells in a 96-well plate. Optical density was measured at 600 nm (OD_600_) every hour during 8 h while incubating the plate at 37°C. Data are presented as means ± standard deviation. No significant statistical differences were observed (*P* > 0.05).

Demonstrating that there is no negative impact of enterobactin depletion on IL-8 expression, PMN migration, and activation in an *in vitro* setting, it was subsequently investigated whether the fitness of *E. coli* was hindered by Ent deficiency *in vitro*. *Escherichia coli* P4 and P4 Δ*entA* were separately grown in DMEM/F12 under the same experimental conditions, and the OD_600_ was measured every hour during 8 h. Both bacteria showed a similar growth curve and no significant differences were observed (Figure 4). This observation therefore indicates *E. coli* P4 Δ*entA* has no reduced fitness relative to *E. coli* P4 under the determined experimental conditions.

### P4 Δ*entA*-Infected Murine Mammary Glands Show Lower Bacterial Loads and a Decreased Inflammatory Response Compared With P4-Infected Glands

**Figure 5:**
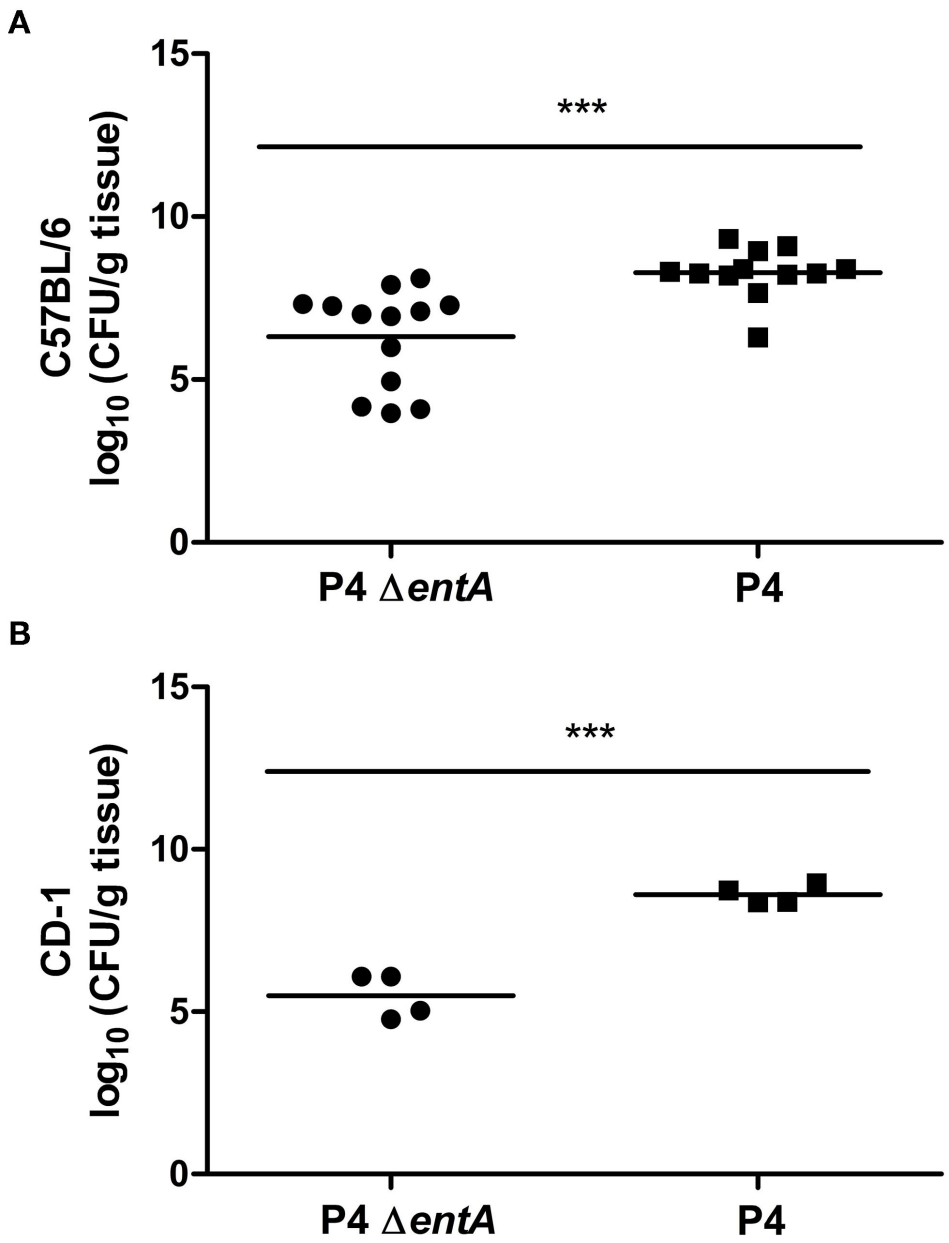
Bacterial load expressed as CFU/g mammary gland in C57BL/6 **(A)** (*n* ≥ 12) and CD-1 **(B)** (*n* = 4) mice at 24 h.p.i. with *E. coli* P4 Δ*entA* (dots) vs. P4 (squares). Data are presented as single sample values. The bar indicates the mean. ***P < 0.001.

**Figure 6:**
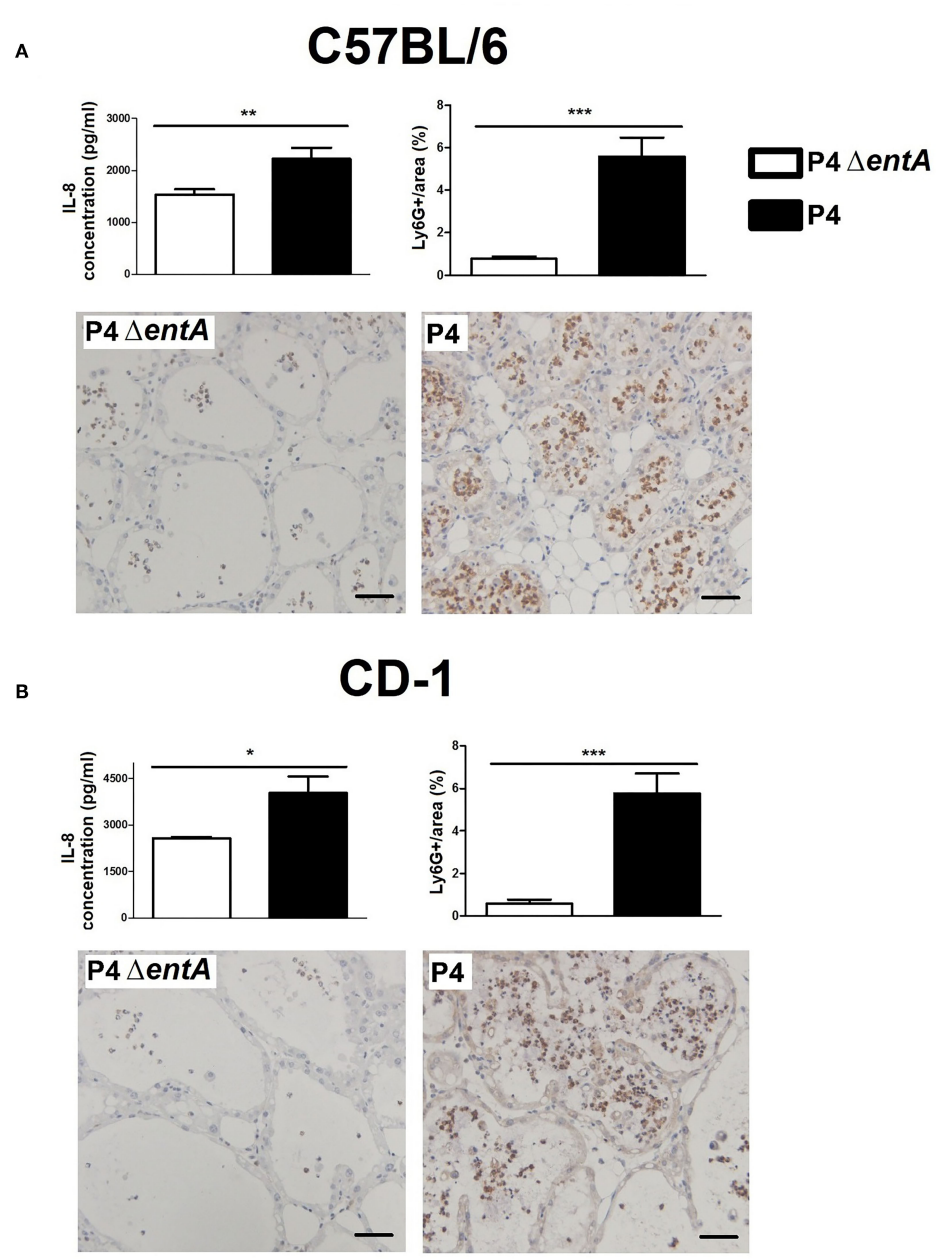
Concentration of the PMN chemoattractant IL-8 and immunohistochemical staining of PMN (Ly6G) in mammary glands of C57BL/6 **(A)** (*n* ≥ 12) and CD-1 **(B)** (*n* = 4) mice at 24 h.p.i. with *E. coli* P4 Δ*entA* vs. P4. Scale bar = 50 μm. Data are presented as means ± standard error of the mean. *P < 0.05; **P < 0.01; ***P < 0.001.

**Figure 7:**
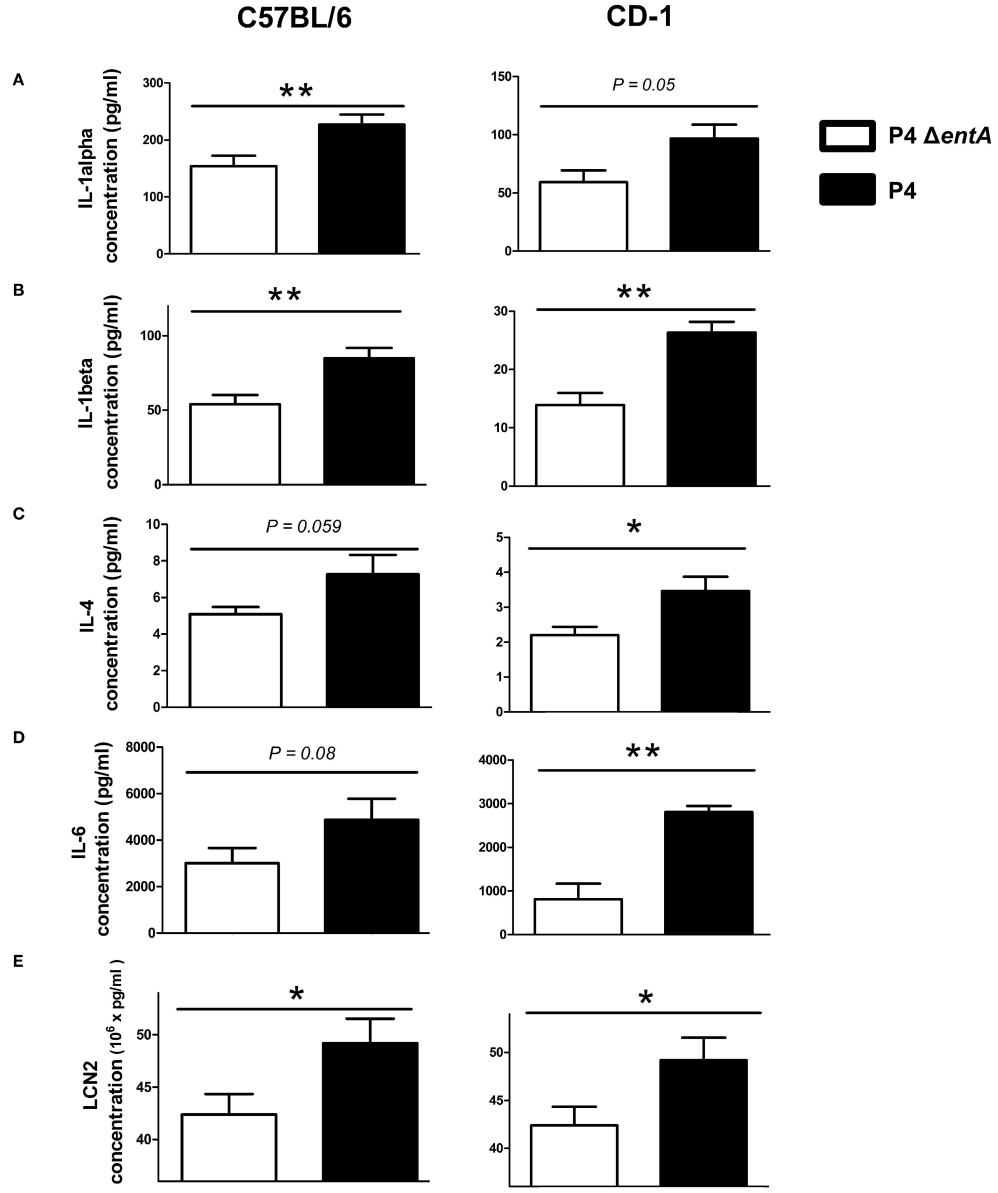
Levels of pro-inflammatory cytokines IL-1α **(A)**, IL-1β **(B)**, IL-4 **(C)**, and IL-6 **(D)** and of LCN2 **(E)** in mammary glands of C57BL/6 (*n* ≥ 12) and CD-1 (*n* = 4) mice at 24 h.p.i. with *E. coli* P4 Δ*entA* vs. P4. Data are presented as means ± standard error of the mean. *P < 0.05 and **P < 0.01.

To investigate the influence of Ent depletion on *E. coli* growth *in vivo*, a situation in which iron availability is more tightly regulated, murine mammary glands were intraductally inoculated with *E. coli* P4 and P4 Δ*entA*. Bacterial loads in P4 Δ*entA*-infected mammary glands were significantly decreased compared with P4-infected glands at 24 h.p.i. (*P* < 0.001; Figure 5). This observation was mouse strain-independent as consistent observations were obtained in both C57BL/6 and CD-1 mouse strains (Figure 5). Concomitant with the reduced IL-8 levels analyzed in mammary gland homogenates, Ly6G staining of mammary gland tissue showed a significantly decreased influx of PMN in the alveolar lumen upon mammary gland infection with P4 Δ*entA* compared with that seen in P4-infected mammary glands (*P* < 0.001; Figure 6). Similar to the reduced levels of IL-8, levels of pro-inflammatory cytokines IL-1α,−1β,−4, and−6 as well as LCN2 were also reduced upon P4 Δ*entA* infection in mammary gland homogenates of both mouse strains compared with their P4-infected counterparts (*P* ≤ 0.08; Figure 7).

## Discussion

Due to the high prevalence of bovine mastitis and the consecutive overuse of antibiotics in the dairy industry, novel compounds that target other determinants such as bacterial fitness factors are desired (16, 35). Limiting iron uptake of *E. coli*, regarded an essential bacterial growth factor, could offer such antimicrobial potential (14, 37). In this preliminary host–pathogen interaction study, we demonstrated the potential of decreasing the fitness of *E. coli* in the murine mammary gland if *E. coli* is Ent deficient. This decreased fitness was observed with significantly reduced bacterial loads in murine mammary gland lysates and, consequently, a decreased inflammatory response which was characterized by diminished chemokine levels and less infiltration of PMN.

Contradictory to our *in vivo* observations, we demonstrated that bacterial Ent depletion did not impact on IL-8 secretion by the bovine mammary epithelium and its associated PMN migration *in vitro*. It must be noted that these experiments were all performed in DMEM/F12, implying there was most likely no significant difference in bacterial growth between *E. coli* P4 Δ*entA* and its P4 wild-type counterpart, because both bacteria showed similar growth patterns in this culture medium. It is therefore highly likely that this similar *in vitro* bacterial growth is responsible for the absence of differences observed in our other *in vitro* assays. Performing these assays again under iron-restricted conditions might reveal novel data that more closely mimic the outcomes of our murine model, and as such, this should be regarded a follow-up experiment. In fact, *E. coli* features more mechanisms than just Ent to acquire iron, which might have compensated for the Ent deficiency of *E. coli* P4 Δ*entA* when grown in high iron containing DMEM/F12 (1, 3, 4). To proceed with our *in vitro* observations, it was also noticed that PMN might have significantly improved phagocytosis and enzymatic activity in their phagosomes if *E. coli* is Ent deficient, although more research remains warranted to fully confirm this statement. Nonetheless, a higher ROS production by PMN in the *E. coli* P4 Δ*entA-*infected murine mammary glands is another possible factor that might have contributed to the observed differences between the *E. coli* P4 Δ*entA* vs. P4-infected mouse mammary glands. Furthermore, it should also be noticed that our *in vitro* research was limited to the interplay between boMECs and PMN. Our goal in this preliminary study was to evaluate the presence of Ent on the established biomarkers of mastitis, primarily IL-8 produced by boMECs and its associated PMN recruitment and activation (38). However, also other cell types such as macrophages are known to play a crucial role in the latter mentioned processes (38, 39). A previous study of our group showed that macrophages are major secretors of LCN2 upon mammary gland infection (39). Including macrophages to these *in vitro* assays, which can also be proposed as a follow-up experiment, could drastically change *E. coli* growth, IL-8 levels, and concomitant PMN recruitment and activation. Taken together, (1) the high iron availability *in vitro*, (2) a higher ROS production by PMN in *E. coli* P4 Δ*entA*, and (3) the presence of macrophages in the murine mammary gland are factors that most likely contributed to the contradictory observations between our *in vitro* and *in vivo* data.

In comparison, another study also reports the reduced bacterial fitness of an *E. coli* P4 *fec* deletion mutant (*E. coli* P4 Δ*fec*), which is an iron-acquiring mechanism to capture ferric citrate (10). This *fec*-lacking *E. coli* P4 mutant was not capable anymore to induce mastitis in cows in comparison with the *E. coli* P4 wild type (10). Our *E. coli* P4 Δ*entA* was challenged at a similar CFU/ml in mice and was still able to establish infection, but significantly lower bacterial loads were observed. Our findings in mice are therefore in line with the observations made in the bovine target species, although it should be noticed that a direct comparison between these two *E. coli* that are deficient in different iron acquisition mechanisms and were evaluated in different species remains difficult. Therefore, a follow-up study in which cows are challenged with *E. coli* P4 Δ*entA* urges to allow a correct comparison between *E. coli* P4 Δ*entA* and *E. coli* P4 Δ*fec*. Nonetheless, our preliminary results in mice together with the observations made for *E. coli* P4 Δ*fec* in dairy cattle pinpoint toward the potential of decreasing *E. coli* viability in the mammary gland by targeting *E. coli*'s iron acquisition mechanisms.

In conclusion, the inability of *E. coli* to produce Ent results in a decreased bacterial fitness of *E. coli* in the murine mastitic mammary gland by a reduced bacterial load and, consequently, a diminished associated inflammatory response. Our study therefore identifies Ent interference as a potential target to decrease *E. coli* growth in the mastitic mammary gland, although several *in vitro* follow-up experiments remain to fully understand the complex interaction between involvement of iron availability, immunity, and an *E. coli* infection. In addition, future research in dairy cattle remains necessary to assess if interference with bacterial Ent indeed has antimicrobial potential in the bovine udder.

## Data Availability Statement

The raw data supporting the conclusions of this article will be made available by the authors, without undue reservation.

## Ethics Statement

The animal study was reviewed and approved by The Regional Government Ethics Committee of Upper Bavaria, Germany (Approval Number: 55.2-1-54-2531-108-05) and the ethics committee of the Faculty of Veterinary Medicine, Ghent University, Belgium (Approval Number: 2015/127).

## Author Contributions

All authors listed have made a substantial, direct and intellectual contribution to the work, and approved it for publication.

## Conflict of Interest

The authors declare that the research was conducted in the absence of any commercial or financial relationships that could be construed as a potential conflict of interest.
