# Supplementary material for: Enterobactin Deficiency in a Coliform Mastitis Isolate Decreases Its Fitness in a Murine Model: A Preliminary Host–Pathogen Interaction Study
Source: Front Vet Sci. 2020 Nov 9;7:576583. doi: 10.3389/fvets.2020.576583 (PMC7680728; doi:10.3389/fvets.2020.576583)
Supplement: Supplementary file 1 [file Table_1.DOCX]

| **Gene** | **Forward primer sequence 5’-3’** | **Reverse primer sequence 5’-3’** |
| --- | --- | --- |
| LCN2 | TCTGTTGGAGGATGGAACTTCG | TAACAGGATGGAGGTGACGTTG |
| 18s | CGGGGAGGTAGTGACGAAA | CCGCTCCCAAGATCCAACTA |
| PPIA | TCCGGGATTTATGTGCCAGGG | GCTTGCCATCCAACCACTCAG |

**Supplemental Table 1:** Primers used for RT-qPCR after reverse transcription of mRNA, derived 24 h.p.i. from bovine udder quarters experimentally infected with 500 CFU *E. coli* 1303.
